# Chromosomal instability in a patient with ring chromosome 14 syndrome: a case report

**DOI:** 10.1186/s13039-024-00686-0

**Published:** 2024-07-18

**Authors:** Juan Pablo Meza-Espinoza, Juan Ramón González-García, Nayeli Nieto-Marín, Liliana Itzel Patrón-Baro, Rosa María González-Arreola, Eliakym Arámbula-Meraz, Julio Benítez-Pascual, Alberto Kousuke De la Herrán-Arita, Claudia Desireé Norzagaray-Valenzuela, Marco Antonio Valdez-Flores, Tomás Adrián Carrillo-Cázares, Verónica Judith Picos-Cárdenas

**Affiliations:** 1 Facultad de Medicina Matamoros, Universidad Autónoma de Tamaulipas, Matamoros, Tamps México; 2 División de Genética, Centro de Investigación Biomédica de Occidente, Instituto Mexicano del Seguro Social (IMSS), Guadalajara, Jalisco México; 3 Maestría en Ciencias en Biomedicina Molecular, Facultad de Medicina, Universidad Autónoma de Sinaloa, Culiacán, Sin México; 4 Doctorado en Genética Humana, Universidad de Guadalajara, Guadalajara, Jalisco México; 5 Facultad de Ciencias Químico-Biológicas, Universidad Autónoma de Sinaloa, Culiacán, Sin México; 6 Facultad de Odontología, Universidad Autónoma de Sinaloa, Culiacán, Sin México; 7 Facultad de Medicina, Universidad Autónoma de Sinaloa, Culiacán, Sin México; 8 Facultad de Biología, Universidad Autónoma de Sinaloa, Culiacán, Sin México

**Keywords:** Ring chromosome 14 syndrome, Chromosomal instability, Dynamic mosaicism

## Abstract

**Background:**

Ring chromosome 14 syndrome is a rare disorder primarily marked by early-onset epilepsy, microcephaly, distinctive craniofacial features, hypotonia, intellectual disability, and delay in both development and language acquisition.

**Case presentation:**

A 21-year-old woman with a history of epileptic seizures since the age of 1.5 years presented with distinctive craniofacial features, including a prominent and narrow forehead, sparse and short eyebrows, palpebral ptosis, horizontal palpebral fissures, a broad nasal bridge, a prominent nasal tip, a flat philtrum, hypertelorism, midfacial hypoplasia, horizontal labial fissures, a thin upper lip, crowded teeth, an ogival palate, retrognathia, and a wide neck. Additional physical abnormalities included kyphosis, lumbar scoliosis, pectus carinatum, cubitus valgus, thenar and hypothenar hypoplasia, bilateral hallux valgus, shortening of the Achilles tendon on the left foot, and hypoplasia of the labia minora. Chromosomal analysis identified a ring 14 chromosome with breakpoints in p11 and q32.33. An aCGH study revealed a ~ 1.7 Mb deletion on chromosome 14qter, encompassing 23 genes. Genomic instability was evidenced by the presence of micronuclei and aneuploidies involving the ring and other chromosomes.

**Conclusion:**

The clinical features of our patient closely resembled those observed in other individuals with ring chromosome 14 syndrome. The most important point was that we were able to verify an instability of the r(14) chromosome, mainly involving anaphasic lags and its exclusion from the nucleus in the form of a micronucleus.

## Background

Human ring chromosomes are structural abnormalities resulting from two breaks in the DNA strand that fuse to form a circular DNA molecule, often leading to genetic material loss. These chromosomes can possess one or more centromeres or lack one entirely, affecting their segregation during anaphase. Ring chromosomes have been reported for all human chromosomes, with approximately half involving acrocentric chromosomes, including chromosome 14 [1]. Numerous cases of chromosome 14 rings have been documented, with the deleted region’s size ranging from 0.3 to 5 megabases (Mb) [2]. The severity of the phenotype, known as ring chromosome 14 syndrome [r(14) syndrome, Online Mendelian Inheritance in Man (OMIM) #616,606], varies among patients [3]. Key clinical features include early-onset epilepsy, microcephaly, hypotonia, intellectual disability, developmental and language delays, and distinctive craniofacial dysmorphisms such as hypertelorism, micrognathia, a thin upper lip, down-turned mouth corners, a high-arched palate, and large, low-set ears [4]. Additional features include motor skill issues, retinal abnormalities, feeding difficulties, and behavioral disorders [2, 3, 5]. The deletion of specific genes on 14q32.2-32.3 is believed to cause the primary clinical features of the syndrome [3], although other genes outside the deleted region might also influence the phenotype [6].

Ring chromosomes exhibit inherent instability due to the sister chromatid exchange process, which can create dicentric or interlocked ring chromosomes. During mitosis, these chromosomes can undergo anaphase lagging, nondisjunction, or fragmentation, leading to cells without the ring chromosome, cells with multiple ring chromosomes, binucleated cells, internuclear bridges, nuclear protrusions, and micronuclei (MN). This phenomenon is known as tissue-specific dynamic mosaicism [7]. Ring chromosomes show variable instability in vivo based on their size and genetic content [8], but there is no clear correlation between the size of a ring chromosome, the occurrence of dynamic mosaicism, and clinical severity [9].

In this report, we present the case of a female with a ring chromosome 14, who exhibited epileptic seizures, craniofacial dysmorphism, skeletal abnormalities, and genital alterations. Chromosomal instability was also observed through the presence of MN and aneuploidies.

## Case presentation

A 21-year-old woman, the fifth child of healthy, unrelated parents, experienced epileptic seizures beginning at 1.5 years old, which were managed with valproic acid. She has been seizure-free since the age of 2 and has been off treatment since the age of 8. Currently, she has a height of 162 cm (50th percentile) and a head circumference of 53.5 cm (10th-25th percentile), along with a high anterior hairline. She exhibits craniofacial dysmorphism, including a prominent and narrow forehead, sparse and short eyebrows, palpebral ptosis, horizontal palpebral fissures, a broad nasal bridge, a prominent nasal tip, a flat philtrum, hypertelorism, midfacial hypoplasia, horizontal labial fissures, a thin upper lip, crowded teeth, an ogival palate, retrognathia, and a wide neck. Additional features include kyphosis, lumbar scoliosis, bilateral cubitus valgus, thenar and hypothenar hypoplasia, bilateral hallux valgus, shortening of the Achilles tendon on the left foot, and hypoplasia of the labia minora. Although clinical images are not provided due to lack of parental authorization, her clinical characteristics align with those typically reported for ring chromosome 14 syndrome (Table 1).

### Conventional cytogenetic studies

**Fig. 1:**
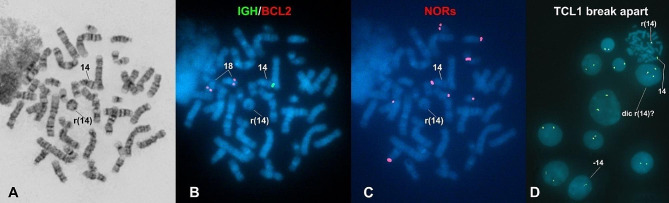
Cells from the patient cultured, harvested, and stained using standard methods for karyotype analysis. **A** GTG-banded metaphase showing the r(14) chromosome. **B** The same metaphase was sequentially analyzed by FISH with IGH/BCL2 probes, revealing that the r(14) chromosome lacks the IGH signal. **C** FISH with NORs probes showed that the r(14) chromosome lacks NORs signals. **D** FISH with the TCL1 probe indicated that the deleted region is distal to the *TCL1* gene. Additionally, two abnormal cells were identified: one with three signals, likely indicating a dicentric r(14), and one with only one signal, suggesting a loss of the r(14) chromosome

Peripheral blood lymphocytes from the patient were cultured and harvested using standard methods for karyotype analysis. Chromosomal analysis of GTG-banded metaphases revealed the presence of a ring chromosome 14 (Fig. 1A). Both parents had a normal karyotype. FISH (fluorescense in situ hybridization) analysis using the TCL1 break-apart and IGH/BCL2 probes indicated the breakpoint occurred distal to the *TCL1* (q32.13) but proximal to the *IGH* (q32.33) genes (Fig. 1B and D). Additionally, FISH with a nucleolus organizer region (NOR) specific probe showed no signal in the r(14) chromosome (Fig. 1C). The frequencies of cells with TCL1 break-apart signals in interphase FISH were as follows: 198/243 cells (81.5%) had two signals, 30/243 cells (12.3%) had only one signal, suggesting the loss of the r(14) chromosome, and 15/243 cells (6.2%) had three signals, likely indicating a duplicated dicentric r(14) chromosome.

### Microarray study

**Fig. 2:**
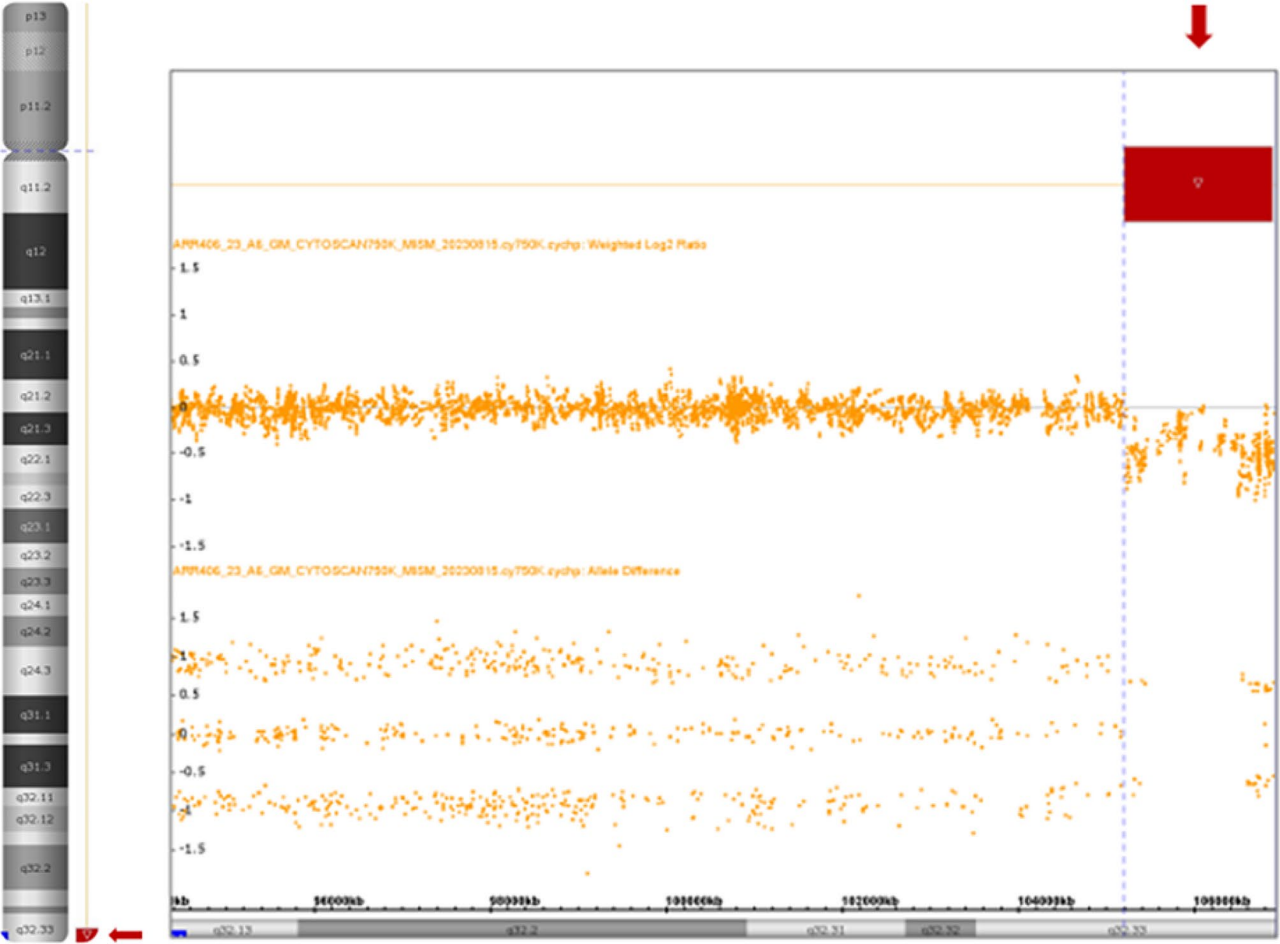
Analysis of aCGH. Left, image representing chromosome 14. Right, plot of weighted log2 ratio. The red arrows indicates the deleted region: arr[GRCh38] 14q32.33 (105,194,385_106,876,229)x1

To determine the extent of the genomic imbalance, array comparative genomic hybridization (aCGH) was performed using CytoScan™ Technology (Thermo Fisher Scientific Inc). The processes of digestion, ligation, Polymerase chain reaction (PCR), purification of PCR products, quantification, fragmentation, labeling, matrix hybridization, washing, staining, and scanning arrays were carried out following the supplier’s recommendations. Data analysis was conducted with ChAS 4.3 software. Results were interpreted using several databases, including the Database of Genomic Variants, Cytogenomics, Array Group CNV Database, Ensembl Resources, OMIM, UCSC Genome Browser, ClinGen, ClinVar, and CHD wiki. The analysis revealed a terminal deletion of approximately 1.7 Mb on chromosome 14, encompassing 23 genes: *BRF1, BTBD6, PACS2, TEX22, MTA1, CRIP2, CRIP1, TEDC1, IGH, TMEM121, LOC105370697, MIR8071-1, MIR8071-2, ELK2, MIR4539, MIR4507, MIR4538, MIR4537, FAM30A, ADAM6, LINC00226, LINC00221*, and *MIR5195* (Fig. 2).

Based on all the studies, the patient’s karyotype was concluded to be 46,XX, r(14).ish r(14)(p11q32.33) (NOR-,TCL1+,IGH-).arr[GRCh38] 14q32.33(105,194,385_106,876,229)x1 dn.

### Ring mitotic instability

**Fig. 3:**
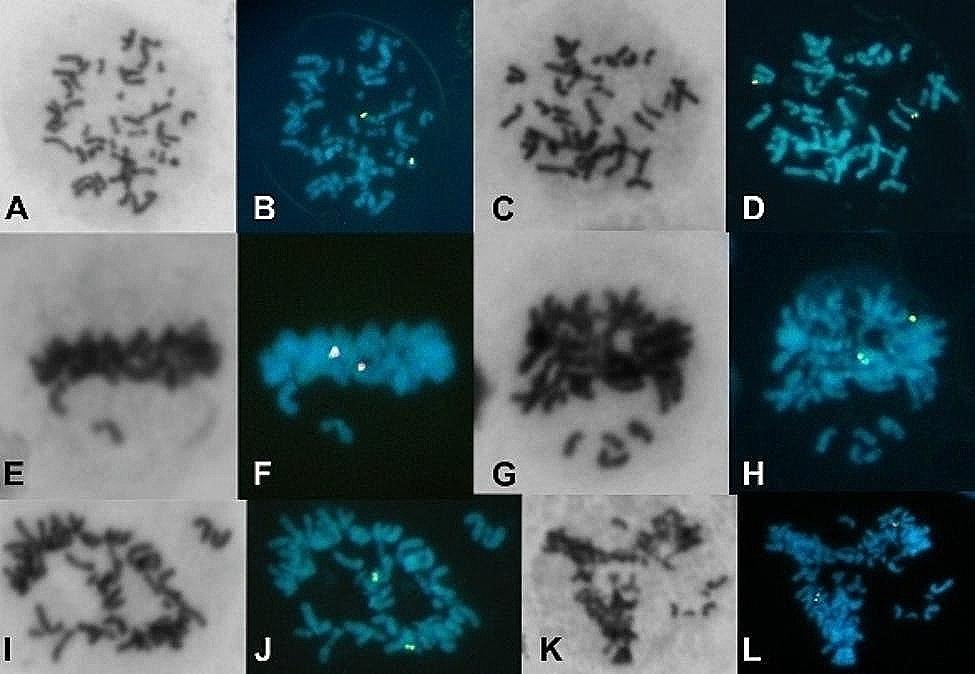
Giemsa-stained altered mitotic cells, sequentially studied by FISH using the TCL1 break-apart probe. These cells were harvested without colchicine block and KCl shock. **A-D** Two metaphase cells with chromosome compaction and distribution resembling those harvested with colchicine. **E-H** Metaphase cells displaying lagging chromosomes, excluding chromosome 14 and r(14). **I-L** Mitotic cells with multipolar-like configurations, also showing lagging chromosomes

Given the known mitotic instability of several ring chromosomes, we investigated this phenomenon in our patient. A new culture of the patient’s peripheral blood lymphocytes, stimulated with phytohaemagglutinin, was prepared. Unlike the standard culture used for karyotype analysis, this culture was directly fixed and washed in a cold solution of absolute methanol: glacial acetic acid (3:1) after 72 h of incubation, without exposure to colchicine or hypotonic shock. Cells suspended in fixative solution were dropped onto microscope slides and stained with Giemsa. Cells in metaphase, anaphase, telophase, or cytokinesis were analyzed for mitotic disturbances, and micronucleated cells were counted. Selected microscopic coordinates were recorded for subsequent FISH analyses using IGH and TCL1 break-apart probes. This method revealed various mitotic disturbances. Several cells failed to align at the classical metaphase plate, showing chromosome compaction and distribution similar to those in a classical colchicine block (Fig. 3A-D). Additionally, some cells displayed chromosome laggards affecting chromosomes other than the r(14) (Fig. 3E-H) or exhibited a multipolar mitosis-like chromosome organization (Fig. 3I-L) (for comparison, see Barajas-Torres et al., 2016 [10]).

**Fig. 4:**
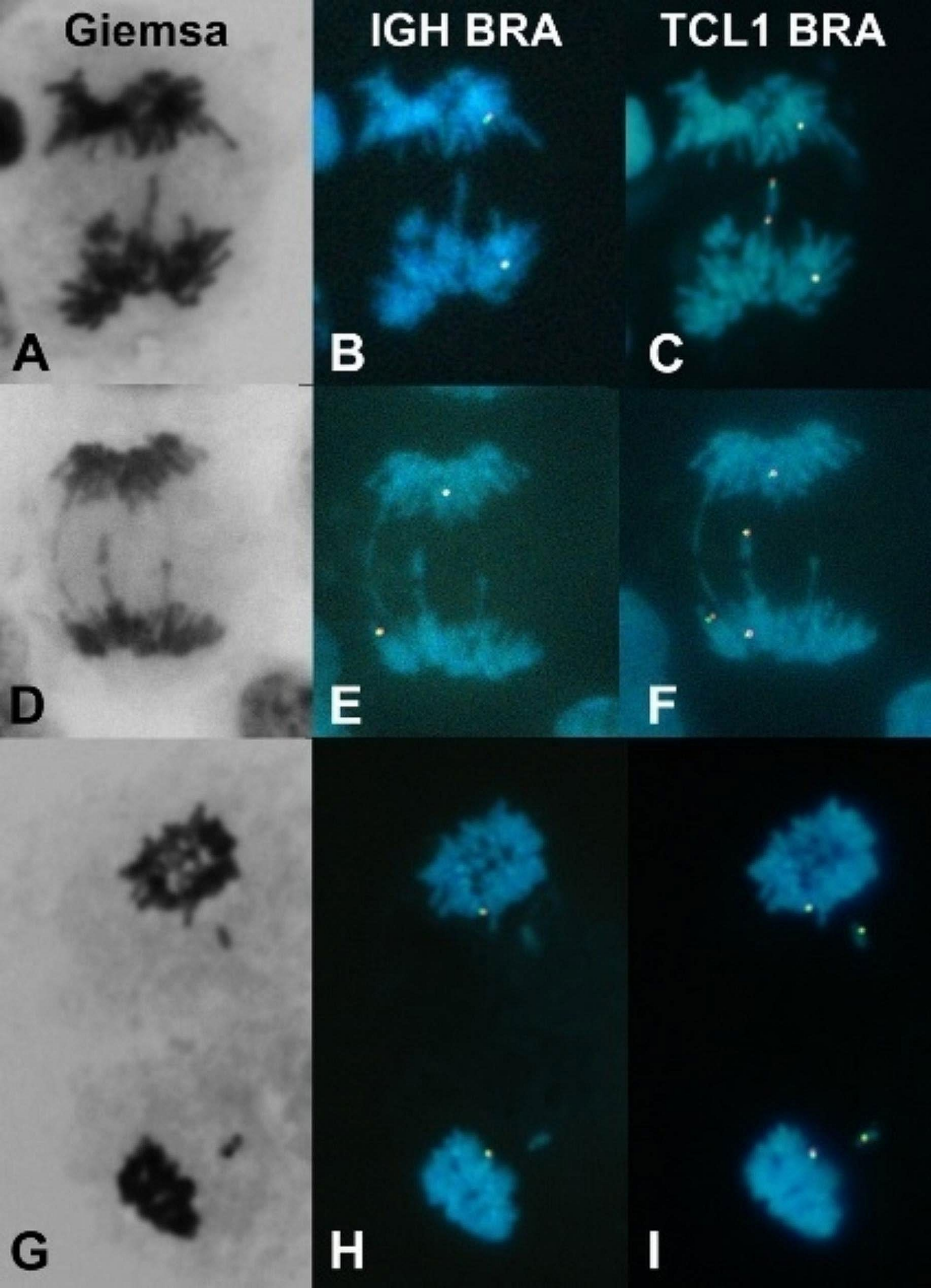
Giemsa-stained mitotic cells serially studied by consecutive FISH with IGH and TCL1 break apart probes (these cells were harvested without both colchicine block and hypotonic shock). **A-C** Anaphase cell in which a dicentric (duplicated) r(14) is in the middle of the mitotic spindle due probably to the nullification of forces generated by the centromeric traction to opposite poles. **D-F** Anaphasic r(14) lagging probably caused by a merotelic union. **G-I** Telophasic cell with r(14) chromosome lagging in both daughter nuclei which, probably, will be a micronucleus in each daughter cell

Sequential FISH assays demonstrated that the segregation of the r(14) chromosome was preferentially affected (Fig. 4: B-C, E-F, and H-I). As a result of this anomalous anaphase-telophase segregation, the r(14) chromosome would be excluded from the main nucleus in the daughter cells, likely forming a micronucleus. The analysis of mitotic figures revealed the following counts of abnormal cells: 33 out of 81 (41%) metaphase cells, 2 out of 10 (20%) anaphase cells, and 4 out of 28 (14%) telophase-cytokinesis cells.

**Fig. 5:**
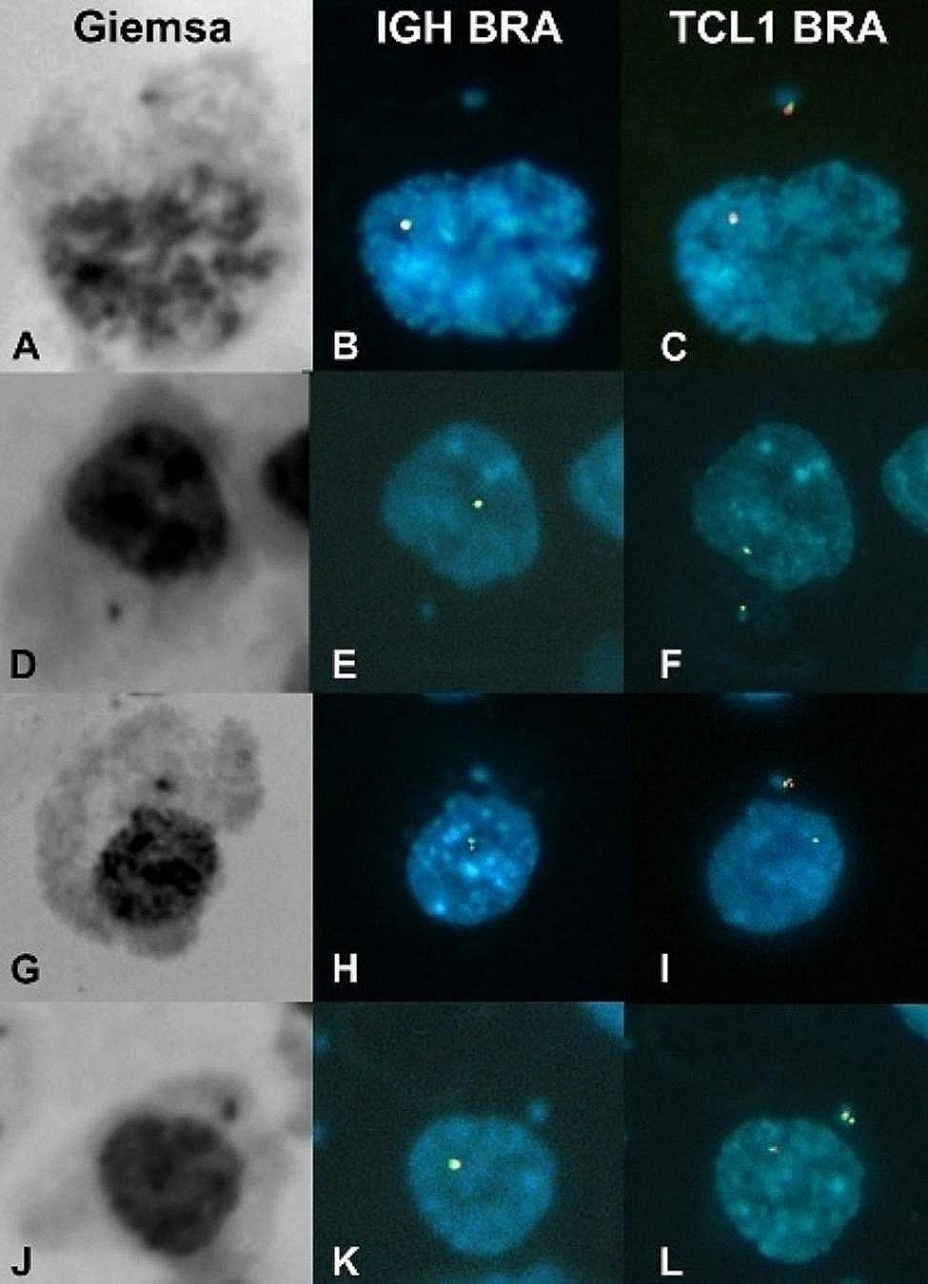
Selected micronucleated cells studied by consecutive FISH with the IGH and TCL1 break apart probes. **A, D, G, J** Giemsa-stained micronucleated cells. **B, E, H, K** Here, micronucleated cells show only one signal of the *IGH* gene located in the normal chromosome 14. Whereas, in **C, F, I, L** these cells show two signals of the *TCL1* gene; interestingly, one of these signals is in the micronucleus of each cell, thus demonstrating that the micronucleus contains the genetic material of the r(14) chromosome. Furthermore, it is noteworthy that MN in I and L show double TCL1 signal, suggesting the presence of a dicentric (duplicated) ring chromosome

Consistent with these findings, we observed MN in 27 out of 305 (9%) interphase cells. FISH analysis of 21 out of those 27 micronucleated cells confirmed the presence of the r(14) chromosome in all of them (Fig. 5).

## Discussion and conclusions

Our patient exhibited typical clinical features of ring chromosome 14 syndrome, including epileptic seizures, craniofacial dysmorphism, and skeletal abnormalities. In 23 reported cases with pure genomic deletions ranging from 0.3 to 5 Mb, including ours (Table 1), a consistent phenotype was observed, particularly with epileptic seizures (23/23), intellectual disability (23/23), microcephaly (22/22), speech impairment (22/23), hypotonia (19/21), and facial dysmorphism (17/23). However, other clinical features such as scoliosis (10/18), ocular anomalies (11/20), and susceptibility to infections (10/19) were also present although at a lower frequency.

**Table 1:** Clinical findings and genomic loss in 23 patients with r(14) syndrome*

Case	Sex/Age	del	del Mb	aCGH NCBI36/HG18	ES	ID	Mc	SI	Hy	FD	Sc	EA	IS
21	M/8	14q32.3	0.3	(106,072,471 − 106,329,869)x1	+	+	+	+	+	-	-	NA	+
6	M/10	14q32.3	0.3	(106,072,471 − 106,329,869)x1	+	+	+	+	+	-	+	-	+
8	F/23	14q32.3	0.65	NA	+	+	+	+	+	-	+	+	+
1	F/17	14q32.3	0.65	(105,717,236 − 106,329,869)x1	+	+	+	+	+	-	-	+	+
20	F/2	14q32.3	0.65	(105,717,236 − 106,329,869)x1	+	+	+	-	NA	-	-	+	-
13	M/16	14q32.33	1.4	(104,918,795 − 106,329,869)x1	+	+	+	+	+	+	+	-	-
**Ours**	**F/21**	**14q32.33**	**1.7**	(104,746,668 − 106,329,869)x1	**+**	**+**	**+**	**+**	**+**	**+**	**+**	**-**	**-**
2	F/18	14q32.33	2	(104,275,679 − 106,329,869)x1	+	+	+	+	+	+	+	+	+
24	F/28	14q32.33	2.2	(104,527,022–106,329,869)x1	+	+	+	+	+	+	+	NA	+
4	F/13	14q32.33	2.5	NA	+	+	+	+	-	-	+	-	-
12	M/5	14q32.33	2.5	(103,867,349 − 106,329,869)x1	+	+	+	+	-	+	-	+	+
16	M/14	14q32.33	2.5	(103,867,349 − 106,329,869)x1	+	+	+	+	+	+	+	+	-
5	M/4	14q32.33	2.5	(103,867,349 − 106,329,869)x1	+	+	+	+	+	+	-	-	-
15	F/1	14q32.3	2.6	(103,705,675 − 106,329,869)x1	+	+	+	+	+	+	-	-	+
22	F/25	14q32.3	3.1	(103,493,209 − 106,329,869)x1	+	+	+	+	NA	+	NA	+	NA
[11]	M/11	14q32.33	3.2	NA	+	+	+	+	+	+	NA	+	NA
17	F/5	14q32.33	3.8	(102,617,278 − 106,329,869)x1	+	+	+	+	+	+	-	-	-
9	F/2	14q32.33	4.3	(102,319,877 − 106,329,869)x1	+	+	+	+	+	+	+	-	-
[12]	F/1	14q32.31	4.7	(101,635,672 − 106,329,869)x1	+	+	+	+	+	+	NA	+	+
26	F/3	14q32.3	5	(101,371,112 − 106,329,869)x1	+	+	+	+	+	+	+	+	-
14	M/1	14q32.31	5	(101,537,205 − 106,329,869)x1	+	+	+	+	+	+	-	-	+
20	M/27	14q32.31	5	(101,537,205 − 106,329,869)x1	+	+	+	+	+	+	NA	NA	NA
[13]	F/4	14q32.31	5	(101,410,294 − 106,329,869)x1	+	+	NA	+	+	+	NA	+	NA

*All cases except [11, 12], and [13] were taken from Zollino et al. (2012) and are presented by their original codes. **M**: Male. **F**: Female. **del**: Deletion. **ES**: Epileptic seizures. **ID**: Intellectual disability **Mc**: Microcephaly. **SI**: Speech impairment. **Hy**: Hypotonia. **FD**: Facial dysmorphism. **Sc**: Scoliosis. **EA**: Eye abnormalities. **IS**: Infection susceptibility. **NA**: Not available. Genomic coordinates are shown in the Human NCBI36/HG18 assembly only for comparison

Chromosomal instability in our case led to chromosome 14 monosomy in some cells (12.3%) and duplication of r(14) sequences in others (6.2%) (Fig. 2-D). This is similar to what has been observed in rings derived from chromosomes other than 14 [14]. The mechanism behind this co-occurrence involves anaphase segregation after a sister chromatid exchange in the r(14) chromosome [2, 9]. However, other mechanisms such as loss of the ring chromosome due to dicentric chromosome formation or merotelic unions may also contribute to chromosome 14 monosomy.

Loss of the ring chromosome can also be caused by other mechanisms. When a dicentric duplicated r(14) chromosome is pulled towards opposite poles at anaphase, the traction forces will be nullified. The dicentric chromosome will remain in the middle of the equatorial plaque (as exemplified in Fig. 4A-C) giving rise to two daughter cells with monosomy, one of which could retain the dicentric chromosome as a micronucleus (as observed in Fig. 5G-L). Merotelic unions of the r(14) are other mechanisms of origin of monosomy 14; in Fig. 4D-F an r(14) chromatid is lagged in the middle of the anaphase cell, probably due to a merotelic union, and then, that chromatid will be absent from the nucleus of the daughter cell. A potential third mechanism of r(14) loss could be a delayed alignment in the equatorial plane of the r(14) at metaphase, as suggested in the Fig. 4G-I, where both r(14) chromatids are lagged during telophase despite an apparent kinetochore-microtubule amphitelic attachment; subsequently, both daughter cells would lose the r(14) chromosome, becoming monosomic. On the other hand, micronucleated cells were detected with a frequency of 9% (27/305). The most significant finding was that all MN from 21 micronucleated cells tested by FISH studies had r(14) chromosome sequences (Fig. 5).

We have not identified reports of micronucleated cells in patients with an r(14) chromosome. However, evidence from ring chromosomes of other chromosomes indicates a propensity for these rings to be excluded from the main nucleus as MN. Ledbetter et al. [15] observed in a case of r(15) that 5 out of 1,000 cells were micronucleated and exhibited one or two silver NOR signals, suggesting the presence of monocentric and dicentric r(15) chromosomes in these MN. Similarly, Los et al. [16] reported 18 chromosome-derived MN in a patient with r(18). Yip et al. [17] observed MN in 4% of interphase cells and 16% of cells treated with cytochalasin B in a patient with r(3). Using whole chromosome painting (WCP3), they demonstrated chromosome 3 sequences in all MN. Urban et al. [18] reported 2.8% of cells with MN in a patient with r(6), all of which contained chromosome 6 centromeric sequences. In a case of r(7), Mehraein et al. [19] observed 3.5% micronucleated cells and confirmed the presence of chromosome 7 material in these MN using FISH with WCP7 and D7Z1 probes. A comparable finding was reported in a case of r(13), with 1% of micronucleated cells and 40% of these MN containing sequences derived from chromosome 13 [20]. Petter et al. [21] also found MN in three cases of r(13); all exhibiting chromosome 13 signals, as confirmed by WCP13. These findings support the hypothesis that the structure of ring chromosomes inherently leads to their exclusion from the main nucleus as MN. This hypothesis is further supported by Rudd et al. [22], who demonstrated experimentally that small ring chromosomes from chromosomes 17 and X missegregate more than normal chromosomes.

Additionally, 33 out of 81 (41%) metaphase cells exhibited abnormalities such as disorganized chromosome alignment in the equatorial plane, characterized by lagging chromosomes other than r(14), chromatin over-compaction similar to colchicine blockade, and multipolar spindle configurations (Fig. 3). It is unlikely that these abnormalities are solely due to the haploinsufficiency of genes in the deleted region, given that *TEDC1* (tubulin epsilon and delta complex 1, required for centriole stability; Breslow et al. [23]) is the only gene directly related to these findings.

A common feature of ring chromosomes, including r(14), is the absence or reduction of the canonical telomeric sequence TTAGGG. Studies on telomere dynamics in interphase lymphocytes show that telomeres are located near the central nuclear region, whereas during postmitotic assembly, they move to the nuclear periphery [24, 25]. Interestingly, during the G2 phase, telomeres assemble into a disk structure [26, 27]. The impact of this telomere positioning on mitosis has not been studied. If the telomeric disk observed in G2 persisted into mitosis, it could act as an anchor for microtubule-kinetochore attachment, ensuring proper chromosome orientation in the equatorial plane and preventing monotelic, syntelic, and merotelic attachments [28].

A delay in microtubule-kinetochore binding or chromosome misorientation due to the lack of telomeric sequences on ring chromosomes could activate the spindle attachment checkpoint (SAC) [29–31], arresting metaphase progression until the issue is resolved. It has been noted that a single unattached kinetochore can activate the SAC and inhibit mitotic progression [32]. A prolonged prometaphase arrest triggers cellular responses such as apoptosis, DNA damage repair activation [33, 34], telomere instability [35], and affects daughter cell proliferation [36]. Thus, SAC activation could explain the observed instability in many ring chromosome cases, including our r(14) case (Fig. 3), characterized by misaligned chromosomes, over-compacted chromosomes, and multipolar spindle configurations, which disrupt centriole duplication independently of cell-cycle progression [37–39]. The coalescence of extra centrosomes might explain findings by Rivera and Dominguez [40], who observed hypodiploidy (34 to 45 chromosomes) and polyploidy (74 to 96 chromosomes) in a patient with a r(4) chromosome.

In conclusion, ring chromosome syndrome is a pathological entity with significant variability due to factors including the affected chromosome, gene loss, positional effects of genes near breakpoints, and unbalanced segregation following ring chromosome sister chromatid exchange. Although previous work has suggested instability associated with chromosome rings, the evidence has been inconclusive [15–21]. Remarkably, we have demonstrated an instability of the r(14) chromosome, mainly involving anaphasic lags and its exclusion from the nucleus in the form of a micronucleus. Further research into the inherent instability of ring chromosomes is needed to clarify these observations in patients with ring chromosomes.

## Data Availability

No datasets were generated or analysed during the current study.

## Abbreviations

aCGH: Array comparative genomic hybridization
FISH: Fluorescense in situ hybridization
GTG-banding: G-bands by trypsin using Giemsa
Mb: Megabases
MN: Micronuclei
NOR: Nucleolus organizer region
OMIM: Online Mendelian inheritance in man
PCR: Polymerase chain reaction
SAC: Spindle attachment checkpoint
